# Development and Evaluation of Gastroretentive Floating Tablets of an Antihypertensive Drug Using Hydrogenated Cottonseed Oil

**DOI:** 10.1155/2013/137238

**Published:** 2013-12-18

**Authors:** Harshal Ashok Pawar, Pooja Ramchandra Gharat, Rachana Vivek Dhavale, Pooja Rasiklal Joshi, Pushpita Pankajkumar Rakshit

**Affiliations:** ^1^Dr. L. H. Hiranandani College of Pharmacy, Smt. CHM Campus, Opp. Ulhasnagar Railway Station, Ulhasnagar, Maharashtra 421003, India; ^2^SVKM'S NMIMS, Vile Parle (W), Mumbai, Maharashtra 400056, India

## Abstract

The aim of the present work was to develop a gastroretentive floating tablet of Atenolol and investigate the effects of both hydrophilic and hydrophobic retardant on *in vitro* release. Atenolol is an antihypertensive drug with an oral bioavailability of only 50% because of its poor absorption from lower gastrointestinal tract. The floating tablets of Atenolol were prepared to increase the gastric retention, to extend the drug release, and to improve the bioavailability of the drug. The floating tablets were formulated using hydrophilic polymers as Hydroxy propyl methyl cellulose (HPMC K4M and HPMC K15M), hydrophobic retardant as a hydrogenated cottonseed oil (HCSO), and sodium bicarbonate as a gas generating agent to reduce floating lag time. The formulated tablets were evaluated for the quality control tests such as weight variation, hardness, friability, swelling index, floating lag time, and total floating time. The *in vitro* release study of the tablets was performed in 0.1 N HCl as a dissolution media. The results of the present study clearly indicates the promising potential of Atenolol floating system as an alternative to the conventional dosage and other sustained release formulations. The study also revealed the effectiveness of HCSO as retardant in combination with HPMC.

## 1. Introduction

The oral bioavailability of many drugs is limited by their unfavourable physicochemical characteristics or absorption in well-defined part of the gastrointestinal tract (GIT) referred as “absorption window” [1]. Prolonged gastric retention improves bioavailability, reduces drug waste, and improves the solubility for drugs that are less soluble in a high pH environment [2]. Various approaches have been investigated to increase the retention of oral dosage form in the stomach, including floating systems, swelling and expanding systems, bioadhesive systems, modified shape systems, high density systems, and other delayed gastric emptying devices [1].

**Figure 1:**
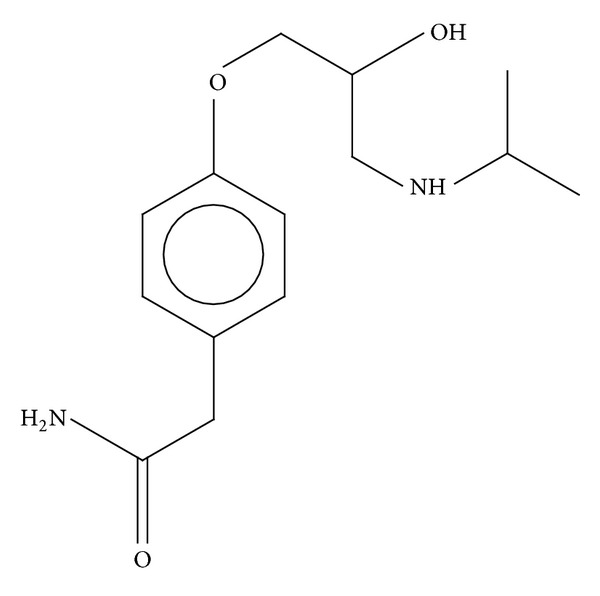
Structure of Atenolol.

Atenolol is a beta (1)-adrenergic antagonist or more commonly known as a beta-blocker used in the treatment of hypertension and angina pectoris. Chemical name of Atenolol is 4-[2-hydroxy-3-[(1-methyl ethyl)amino]propoxy]benzene acetamide. Molecular structure of Atenolol is as shown Figure 1.

Atenolol undergoes little or no hepatic first pass metabolism and its elimination half-life is 6 to 7 hours. The present modes of administration of Atenolol are oral and Parenteral. It is incompletely absorbed from the gastrointestinal tract and has an oral bioavailability of only 50%, while the remaining is excreted unchanged in faeces. Therefore, it is selected as a suitable drug for the design of a gastroretentive floating drug delivery system (GFDDS) with a view to improve its oral bioavailability.

Hydroxy propyl methyl cellulose (HPMC) is hydrophilic cellulose ether widely used as release retarding material. HPMC releases drug by diffusion mechanism. HCSO belongs to USP-NF type 1 consisting of triglycerides of hydroxy stearic acid widely used as a tablet lubricant [3]. In the present study, HCSO was investigated as hydrophobic matrix forming retardant as well as floating material.

The objective of the present study was to develop a gastroretentive floating drug delivery system (GFDDS) of Atenolol and to examine the effects of both hydrophilic and hydrophobic retardant on *in vitro* drug release. In the present study, Atenolol floating tablets were prepared by using hydrophilic polymer, HPMC K4M, HPMC K15M, and HCSO as a hydrophobic retardant, alone and in combination to study the release kinetics and find out the effects of both the retardants and their combinations.

## 2. Materials and Methods

### 2.1. Materials

Atenolol was obtained as a gift sample from Kopran Pvt. Ltd., Mumbai. HPMC K4M and HPMC K15M were supplied by Colorcon Pvt. Ltd., Goa. Hydrogenated cottonseed oil was obtained as a gift sample from Lubritab, New York. All other chemicals and reagent used were of analytical grade.

### 2.2. Drug Excipients Compatibility Study

Compatibility studies were carried out to know the possible interactions between Atenolol and excipients used in the formulation. Physical mixtures of drug and excipients in the ratio 1 : 1 were prepared to study the compatibility. Drug polymer compatibility studies were carried out using FTIR spectroscopy. The IR spectra's were recorded in between 500–4000 cm^−1^.

### 2.3. Preparation of Tablets

Floating tablets containing Atenolol were prepared by direct compression technique using varying concentrations of retardants (HPMC and HCSO) with sodium bicarbonate. All the powders were accurately weighed and passed through 40 mesh sieve. Then, except Magnesium stearate all other ingredients were mixed thoroughly for 15 minutes. After sufficient mixing of drug as well as other components, Magnesium stearate was added, as post lubricant, and the blend was further mixed for additional 2-3 minutes. The final blend was compressed into tablets having average weight of 300 mg using a single punch tablet machine (Royal Artist, India) fitted with an 10 mm round flat punches. The compositions of all formulations are given in Table 1.

### 2.4. Evaluation of Tablet Properties

#### 2.4.1. Determination of Precompression Parameters

The preformulation studies including Bulk density, Tapped density, Hausner's ratio, and Angle of repose were performed of the powder [4].

#### 2.4.2. Determination of Postcompression Parameters

Consider the following.

(*1) Hardness Test.* Monsanto hardness tester was used for the determination of hardness of tablets [5].

(*2) Friability*. Twenty tablets were accurately weighed and placed in the friabilator (Roche's Friabilator) and operated for 100 revolutions. The tablets were dedusted and reweighed. The tablets that loose less than 1% weight were considered to be compliant [6].

The % friability was then calculated by

(1) $$\begin{array}{c} \%\;\text{friability}=\frac{\left(\text{initial}\;\text{weight}-\text{final}\;\text{weight}\right)}{\text{initial}\;\text{weight}}*100. \end{array}$$

(*3) Weight Variation.* Twenty tablets were selected randomly from the lot and weighed individually to check for weight variation [7].

(*4) Drug Content (Assay).* Ten tablets were finely powdered; quantities of the powder equivalent to 50 mg of Atenolol were accurately weighed and transferred to a 100 mL of volumetric flask. The flask was filled with 0.1 N HCl (pH 1.2 buffers) solution and mixed thoroughly. The solution was made up to volume 100 mL and filtered. Dilute 1 mL of the resulting solution to 100 mL with 0.1 N HCl. The absorbance of the resulting solution was measured at 226 nm using a Shimadzu UV-visible spectrophotometer. The linearity equation obtained from calibration curve was used for estimation of Atenolol in the tablet formulations [8].

(*5) In Vitro Buoyancy Studies.* The tablets were placed in a 250 mL beaker, containing 200 mL of 0.1 N HCl. The time required for the tablet to rise to the surface and float was determined as floating lag time (FLT) and the time period up to which the tablet remained buoyant is determined as total floating time (TFT) [9].

(*6) Swelling Study.* The tablets were weighed individually (designated as *W*_0_) and placed separately in petri dish containing 5 mL of 0.1 N HCl and incubated at 37°C ± 1°C. At regular 2 h time intervals until 12 h, the tablets were removed from petri dish, and the excess surface liquid was removed carefully using the tissue paper [10]. The swollen floating tablets were then reweighed (*W*_*t*_) and % swelling index (SI) was calculated using the following formula:

(2) $$\begin{array}{c} \text{SI}\;\left(\%\right)=\left(W_{t}-\frac{W_{0}}{W_{0}}\right)\times 100. \end{array}$$

(*7) In Vitro Dissolution Studies.* The *in vitro *dissolution of all the batches were carried out in 0.1 N HCl as the dissolution medium using USP Type II apparatus (TDT-08L, Electrolab) apparatus at 50 rpm. The temperature was maintained at 37 ± 0.5°C. The dissolution was carried out for 12 hours. The absorbances of the samples at different time intervals were carried out using UV visible spectrophotometer (UV 1800, Shimadzu) at *λ* max of 226 nm [11].

(*8) Kinetics Study.* The mechanism of Atenolol release from the floating tablets was studied by fitting the dissolution data of optimized formulation in following models:

 Zero order: *M* = *M*_0_ − *K*_0_ *t*;
 First order: Log *C* = Log *C*_0_ − *Kt*/2.303;
 Higuchi square root law: *Q* = *kt* ^1/2^;
 Korsemeyer's model: *M*_*t*_/*M*_*∞*_ = *kt* ^*n*^;

where *M*, *C*, and *Q* are the amount of drug released at time *t*, *M*_0_, and *C*_0_ are total amount of drug, and *K*_0_, *K*, and *k* are corresponding rate constant. In case of Korsemeyer's model *M*_*t*_/*M*_*∞*_ is the fractional drug release at time *t*, *k* is a constant incorporating the properties of the macromolecular polymeric systems and the drug, *n* is a kinetic constant, which is used to characterize the transport mechanism. The value of *n* for a cylinder is <0.5 for fickian release, 0.5 < *n* < 1.0 for Anomalous transport (Nonfickian diffusion), 1.0 for Case-II transport, >1.0 for Super Case-II transport type release [12].

(*9) Stability Studies.* The optimized formulation of Atenolol were packed in amber color bottle and aluminum foil laminated on the upper part of the bottle and these packed formulation was stored in stability chamber maintained at 40°C ± 2°C and 75% ± 5% RH for 3 months. The samples were withdrawn periodically and evaluated for their drug content, *in vitro *buoyancy studies and for *in vitro *drug release [13].

## 3. Result and Discussion

### 3.1. Drug-Excipients Compatibility Studies

**Figure 2:**
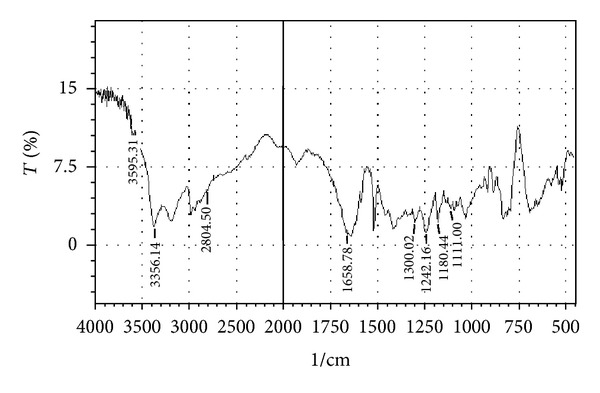
FTIR spectrum of drug (Atenolol).

**Figure 3:**
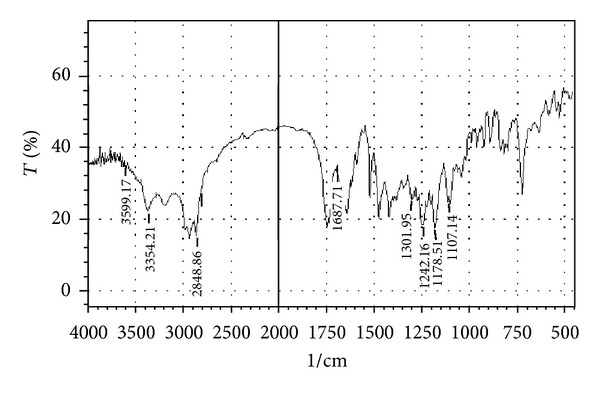
FTIR spectrum of drug with HCSO.

**Figure 4:**
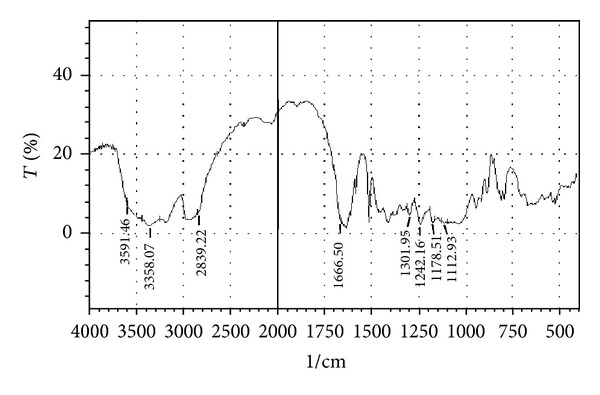
FTIR spectrum of drug with HPMC K4M.

**Figure 5:**
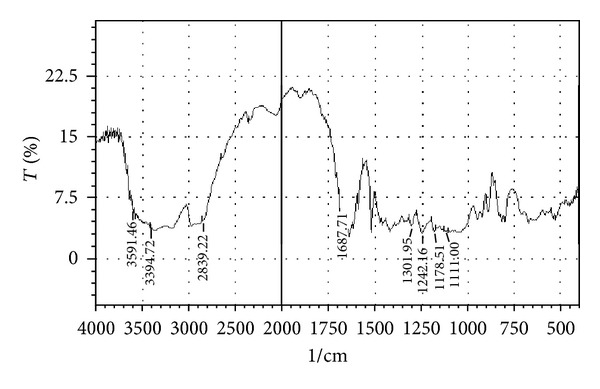
FTIR spectrum of drug with HPMC K15M.

**Figure 6:**
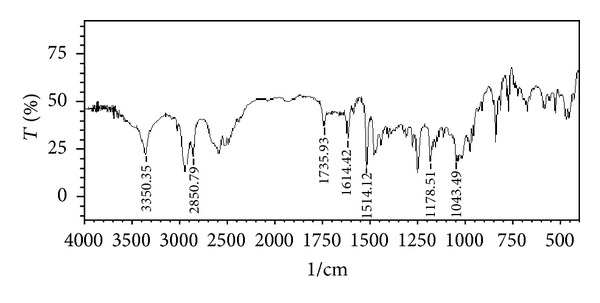
FTIR spectrum of optimized batch (F17).

The peaks obtained in the spectra of each formulation correlates with the peaks of drug spectrum. It does not show any well-defined interaction between Atenolol and excipients. This indicates that the drug is compatible with the formulation components. The spectra for pure drug, drug-excipients mixture and optimized formulation are shown in Figures 2, 3, 4, 5, and 6.

### 3.2. Precompression Parameters

Results of the precompression parameters performed on the blend for batch F1 to F18 are tabulated in Table 2. The bulk density and the tapped density for all the formulations varied from 0.384 to 0.486 g/mL and 0.4809 to 0.5667 g/mL, respectively. The percentage compressibility of powder was determined using Carr's compressibility index. Carr's index lies within the range of 11.2 to 23.08%. All formulations show good compressibility. Hausner ratio was found to be in the range of 1.13 to 1.22. Angle of repose of all the formulations was found to be less than 30°, which indicates a good flow property of the powders.

### 3.3. Postcompression Parameters

The formulated tablets were subjected for post compressional evaluation such as thickness, hardness, weight variation, friability, drug content, *in vitro *buoyancy studies, swelling studies, *in vitro *dissolution studies, and stability studies. Tablet thickness (*n* = 3) was almost uniform in all the formulations and values for tablets ranged from 3.2 to 3.88 mm. The hardness of all formulations was in the range of 8 to 12 kg/cm^2^, indicating satisfactory mechanical strength. The weights of tablets ranged from 290 to 312 mg. All the tablets passed weight variation test as the % weight variation was within the acceptable limits of ±5% of the weight as per Indian Pharmacopoeia. The friability values ranged from 0.11 to 0.49%. All the values are below 1% indicating that the tablets of all formulations are having good compactness and showing enough resistance to the mechanical shock and abrasion. The percent drug content of the tablets was found to be in between 97 to 103%.  Table 3 shows the results of physicochemical characters of Atenolol tablets.

### 3.4. *In Vitro* Buoyancy Studies

*In vitro *buoyancy of the tablets from each formulation F1 to F18 was evaluated and the results are mentioned in Table 4, where the highest and lowest floating lag time (FLT) were observed with the formulation hydrogenated cottonseed oil and HPMC, respectively.

### 3.5. Swelling Index

**Figure 7:**
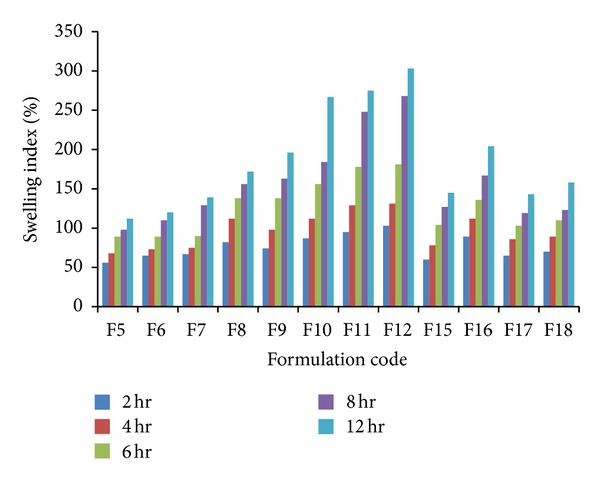
Swelling index of gastroretentive floating tablets of Atenolol.

The swelling index of the tablets from each formulation F1 to F18 was evaluated and the results are mentioned in Table 5 and plot of % swelling index versus time (hrs) is depicted in Figure 7, where the highest and lowest swelling was observed with the formulation F5 and F12 after 12 hrs, respectively. No significant swelling was observed with Formulation F1–F4 since they were prepared using HCSO. The swelling index was increased with concentration of HPMC since this polymer gradually absorbs buffer due to hydrophilic nature.

### 3.6. *In Vitro* Dissolution Studies

**Figure 8:**
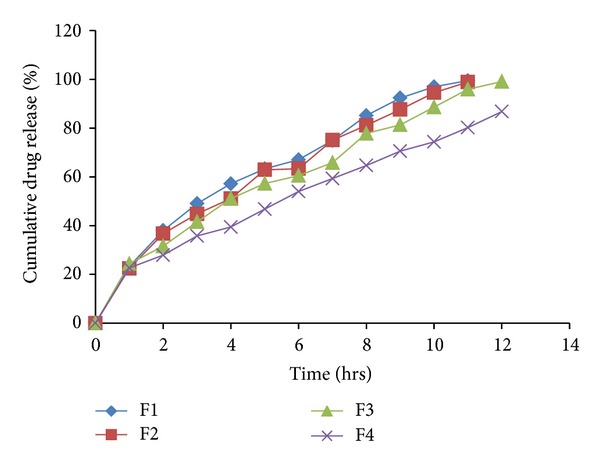
*In vitro* drug released profile of formulations F1 to F4.

**Figure 9:**
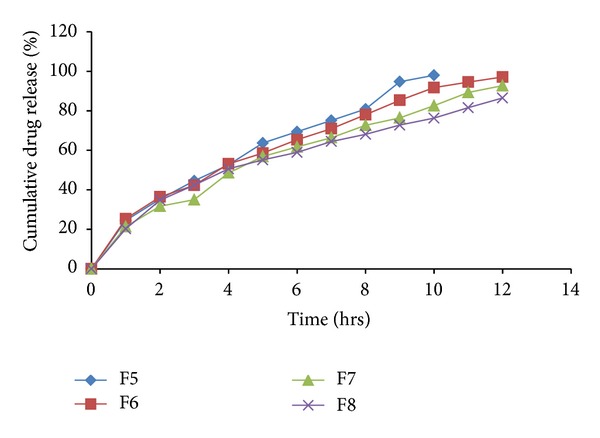
*In vitro* drug released profile of formulations F5 to F8.

**Figure 10:**
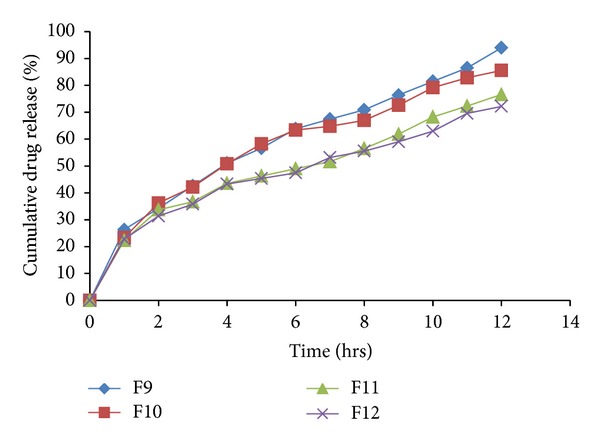
*In vitro *drug released profile of formulations F9 to F12.

**Figure 11:**
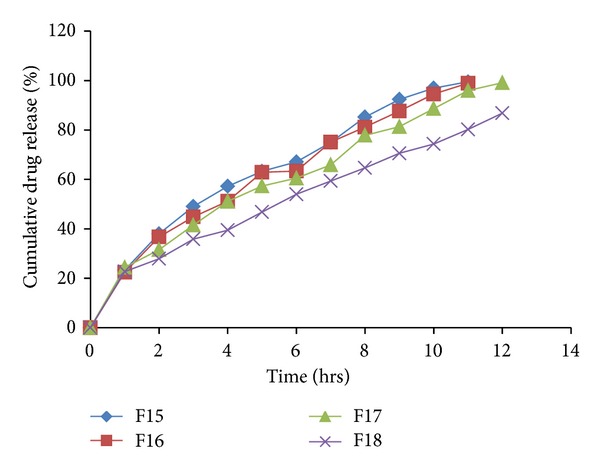
*In vitro *drug released profile of formulations F15 to F18.

The *in vitro *drug release profiles for the formulations F1–F18 were depicted in Tables 6 and 7. The plot of cumulative percentage drug release versus time (hrs) for formulations F1–F4, F5–F8, F9–F12 and F15–F17 were plotted and depicted in Figures 8, 9, 10, and 11, respectively.

Effects of various ingredients and their concentration on drug release were studied. Formulations F1 and F2 showed release of 87.17% and 78.22% at end of 4th hr, respectively. While F3 and F4 showed release of 66.81% and 44.58% at the end of 4th hr, respectively, indicating sustained effect due to higher concentration of HCSO.

Batches formulated with HPMC K4M showed decrease in % drug release with increase in polymer concentration. Formulation F6 showed release of 97.13% at end of 12th hr, while F7 and F8 exhibited higher retardation.

High viscosity grade HPMC contents results in a greater amount of gel being formed. This gel increases diffusion path length of the drug. Its viscous nature also affects the diffusion coefficient of the drug.

As a result reduction in drug release is obtained. Batches formulated with HPMC K15M and HPMC K4M showed similar release pattern till 4th hr. After 4th hr, release was retarded with formulations containing HPMC K15M to higher extent because of its high viscosity as compared to HPMC K4M. Thus, all HPMC K15M formulations exhibited sustained effect for more than 12 hrs.

It was observed that the type of polymer/retardant influences the drug release pattern. HCSO showed release of drug by erosion mechanism, while HPMC by diffusion mechanism. It was observed that as the concentration of polymer/retardant increased in formulations, the % drug release was decreased. Formulations F13 and F14 busted in 1 hr due to failure of matrix to entrap gas.

Formulation F15 with HCSO, HPMC K4M, and HPMC K15M in ratio 2 : 1 : 1 showed release of 57.2% at end of 4th hr. While F16 with HCSO, HPMC K4M, and HPMC K15M in ratio 1 : 2 : 1 showed release of 51.13%, but complete drug release occurred at 11th hr. Formulation F17 was considered as optimized formulation because it showed 51.08% drug release at end of 4th hr and successful sustained effect up to 12 hrs. Formulation F18 with higher amount of hydrophilic polymers, HPMC K4M and HPMC K15M showed release more than 12 hrs. Thus, the optimum combination of hydrophilic-hydrophobic matrix forming material required in formulation to get buoyancy and release of drug over 12 hrs.

### 3.7. Curve Fitting Analysis

The data obtained from *in vitro* dissolution studies were fitted to zero-order, first-order, Higuchi, and Korsemeyer-Peppas equations. The dissolution data obtained were plotted as time versus cumulative percent drug released as zero order, time versus log cumulative percent drug remaining as First order release kinetics, square root of time versus cumulative percent drug released as Higuchi equation, and log time versus log cumulative percent drug released as per Korsemeyer-Peppas equation. The best fit with the highest determination *R* ^2^ coefficients was shown by both Peppas and zero order model followed by Higuchi model which indicate the drug release *via* diffusion mechanism. Zero-order rate equation, which describes the system where release rate is independent of the concentration of the dissolved species. The Korsemeyer-peppas equation is used to analyze the release of pharmaceutical polymeric dosage forms, when the release mechanism is not well known or when more than one type of release phenomena could be involved. The values of *n* with regression coefficient of all the formulations are shown in Table 8. The value of *n* obtained was in the range of 0.519 to 0.765, indicating nonfickian diffusion in case of tablets formulated with HPMC K4M only. While tablets of hydrogenated cotton seed oil and HPMC K15M followed fickian diffusion, matrix tablet of HPMC and hydrogenated cottonseed oil followed nonfickian diffusion. From the results, it was confirmed that all the formulations are following zero order models followed by higuchi model which indicate the drug release *via *diffusion mechanism. Formulation F17 gave 99.08% drug release at 12th hr fulfilling the aim of study and, hence, was selected as optimized batch.

### 3.8. Stability Studies

The results of stability studies did not show any significant change in the physical appearance, drug content, *in vitro *buoyancy studies, and *in vitro* dissolution studies of above four formulations as shown in Table 9.

## 4. Conclusion

The results of the present research work demonstrates that the combination of both hydrophilic and hydrophobic polymers successfully employed for formulating the sustained release matrix tablets of Atenolol. It is observed that optimum concentration of each of the polymer in combination was able to produce desired formulation which releases complete drug in 12 hours. The mechanism of drug release has observed the combined effect of diffusion and erosion for sustained drug release. So, the combination of both hydrophilic and hydrophobic retardant was suitable to produce the matrix tablet rather than using a single type of polymer. The present study also revealed that HCSO can be used as a matrix-forming agent for the preparation of floating tablets. Using HCSO also makes the formulation cost effective.

## Figures and Tables

**Table:** (a)

S. No	Ingredients	F1	F2	F3	F4
1	Atenolol	50	50	50	50
2	Hydrogenated cottonseed oil	50	100	150	175
3	Sodium bicarbonate	45	45	45	45
4	Avicel	152	102	52	27
5	Magnesium stearate	3	3	3	3

**Table:** (b)

S. No	Ingredients	F5	F6	F7	F8
1	Atenolol	50	50	50	50
2	HPMC K4M	50	100	150	175
3	Sodium bicarbonate	45	45	45	45
4	Avicel	152	102	52	27
5	Magnesium stearate	3	3	3	3

**Table:** (c)

S. No	Ingredients	F9	F10	F11	F12
1	Atenolol	50	50	50	50
2	HPMC K15M	50	100	150	175
3	Sodium bicarbonate	45	45	45	45
4	Avicel	152	102	52	27
5	Magnesium stearate	3	3	3	3

**Table:** (d)

S. No	Ingredients	F13	F14	F15	F16	F17	F18
1	Atenolol	50	50	50	50	50	50
2	Hydrogenated cottonseed oil	16.67	33.33	50	25	70	15
3	HPMC K4M	16.67	33.33	25	50	15	70
4	HPMC K15M	16.67	33.33	25	25	15	15
5	Sodium bicarbonate	45	45	45	45	45	45
6	Avicel	152	102	102	102	102	102
7	Magnesium stearate	3	3	3	3	3	3

**Table 2:** Precompression parameters of designed formulations (F1–F17).

Formulation code	Precompression evaluation parameters				
	Bulk density (mg/mL)	Tapped density (mg/mL)	Carr's index	Angle of repose	Hausner's ratio
	(*n* = 3) Mean ± SD	(*n* = 3) Mean ± SD	(%)	(*n* = 3) Mean ± SD	
F1	0.384 ± 0.009	0.5 ± 0.010	23.08	23.01 ± 1.289	1.13
F2	0.4081 ± 0.007	0.5 ± 0.012	18.38	27.02 ± 1.30	1.22
F3	0.4545 ± 0.006	0.52631 ± 0.014	13.64	22.29 ± 0.912	1.55
F4	0.4166 ± 0.010	0.52631 ± 0.011	15.56	28.36 ± 1.344	1.18
F5	0.4233 ± 0.004	0.4825 ± 0.013	12.4	28.1 ± 1.23	1.14
F6	0.4324 ± 0.009	0.5059 ± 0.008	15.7	28.1 ± 1.45	1.17
F7	0.4256 ± 0.008	0.4809 ± 0.015	12.8	27.6 ± 0.82	1.13
F8	0.4065 ± 0.011	0.4837 ± 0.011	15.9	29.32 ± 1.23	1.19
F9	0.4324 ± 0.009	0.4886 ± 0.009	12.8	27.6 ± 1.66	1.13
F10	0.4065 ± 0.007	0.4837 ± 0.012	12.3	29.36 ± 1.25	1.19
F11	0.4546 ± 0.006	0.5227 ± 0.014	11.2	28.76 ± 1.33	1.15
F12	0.4166 ± 0.009	0.4915 ± 0.012	11.3	26.74 ± 0.911	1.18
F13	0.4324 ± 0.011	0.48861 ± 0.009	12.2	25.3 ± 1.155	1.13
F14	0.4088 ± 0.006	0.4782 ± 0.010	11.5	28.7 ± 0.81	1.17
F15	0.4632 ± 0.005	0.528 ± 0.011	14.7	28 ± 1.43	1.14
F16	0.4321 ± 0.009	0.4882 ± 0.012	15.7	29.2 ± 1.234	1.13
F17	0.4776 ± 0.007	0.5667 ± 0.014	16.9	28.67 ± 1.33	1.14
F18	0.486 ± 0.005	0.523 ± 0.012	11.5	27.34 ± 1.55	1.13

**Table 3:** Postcompression parameters of designed formulations (F1–F18).

Formulation code	Postcompression evaluation parameters				
	Thickness (mm)	Hardness (kg/cm)	Weight variation (mg)	Friability (%)	Drug content (%)
	(*n* = 3) Mean ± SD	(*n* = 3) Mean ± SD	(*n* = 20) Mean ± SD	(*n* = 10) Mean ± SD	(*n* = 3) Mean ± SD
F1	3.36 ± 0.08	12 ± 0.08	300.45 ± 1.03	0.11 ± 0.02	98 ± 0.88
F2	3.33 ± 0.02	10 ± 0.05	294.66 ± 0.07	0.49 ± 0.03	101 ± 0.98
F3	3.76 ± 0.06	8 ± 0.07	310.66 ± 0.03	0.58 ± 0.05	99.45 ± 1.45
F4	3.56 ± 0.08	8 ± 0.10	302.33 ± 0.04	0.17 ± 0.04	97.33 ± 0.07
F5	3.22 ± 0.05	12 ± 0.05	305.33 ± 0.06	0.478 ± 0.10	101 ± 0.96
F6	3.54 ± 0.04	12 ± 0.07	298.63 ± 0.05	0.17 ± 0.03	99.7 ± 1.44
F7	3.78 ± 0.08	12 ± 0.06	311.66 ± 0.10	0.166 ± 0.02	98.32 ± 1.27
F8	3.2 ± 0.03	12 ± 0.10	307.66 ± 0.04	0.289 ± 0.05	99.2 ± 0.87
F9	3.6 ± 0.07	12 ± 0.11	291.33 ± 0.08	0.176 ± 0.02	102.3 ± 0.69
F10	3.4 ± 0.05	12 ± 0.04	303.66 ± 0.10	0.16 ± 0.01	99 ± 0.45
F11	3.22 ± 0.06	12 ± 0.05	297.66 ± 0.24	0.162 ± 0.03	97 ± 0.95
F12	3.6 ± 0.04	12 ± 0.07	311.66 ± 0.03	0.152 ± 0.05	101 ± 0.23
F13	3.5 ± 0.03	10 ± 0.10	306.66 ± 0.02	0.127 ± 0.05	101 ± 0.66
F14	3.5 ± 0.09	10 ± 0.03	309.66 ± 0.10	0.133 ± 0.06	99 ± 0.78
F15	3.45 ± 0.10	10 ± 0.07	301.66 ± 0.06	0.117 ± 0.05	97.56 ± 0.98
F16	3.56 ± 0.11	10 ± 0.05	299.33 ± 0.08	0.124 ± 0.07	103 ± 0.95
F17	3.88 ± 0.03	10 ± 0.03	312.12 ± 0.06	0.323 ± 0.04	99.12 ± 1.02
F18	3.45 ± 0.02	10 ± 0.07	290.12 ± 0.03	0.45 ± 0.05	102 ± 0.05

**Table 4:** Floating lag time and total floating time of designed formulations (F1–F18).

S. No	Formulation code	Floating lag time (min:sec)	Total floating time (hrs)
1	F1	3:01	>12
2	F2	2:41	>12
3	F3	2:19	>12
4	F4	2:12	>12
5	F5	2:06	>12
6	F6	1:60	>12
7	F7	1:53	>12
8	F8	1:40	>12
9	F9	1:20	>12
10	F10	1:15	>12
11	F11	1:18	>12
12	F12	1:10	>12
13	F15	1:17	>12
14	F16	1:06	>12
15	F17	1:09	>12
16	F18	1:02	>12

**Table 5:** Swelling Index of gastroretentive floating tablets of Atenolol.

S. No	Formulation code	Swelling index (%) at different time interval				
		2 hr	4 hr	6 hr	8 hr	12 hr
1	F5	56	68	89	98	112
2	F6	65	73	89	110	120
3	F7	67	75	90	129	139
4	F8	82	112	138	156	172
5	F9	74	98	138	163	196
6	F10	87	112	156	184	267
7	F11	95	129	178	248	275
8	F12	103	131	181	268	303
9	F15	60	78	104	127	145
10	F16	89	112	136	167	204
11	F17	65	86	103	119	143
12	F18	70	89	110	123	158

**Table 6:** The *in vitro* drug release profiles for the formulations (F1–F8).

Time (hrs)	F1	F2	F3	F4	F5	F6	F7	F8
0	0	0	0	0	0	0	0	0
1	27.92 ± 0.65	30.23 ± 0.45	24.45 ± 0.90	21.34 ± 0.84	24.44 ± 0.55	25.34 ± 0.56	21.33 ± 0.78	20.22 ± 0.60
2	73.9 ± 0.77	55.65 ± 0.60	33.21 ± 0.67	29.35 ± 0.67	35.31 ± 1.45	36.42 ± 1.34	31.7 ± 0.60	34.75 ± 0.78
3	83.46 ± 0.77	72.05 ± 0.34	41.17 ± 0.45	34.65 ± 0.57	44.54 ± 0.60	42.3 ± 0.45	35.01 ± 0.66	42.37 ± 0.66
4	87.17 ± 0.25	78.22 ± 0.67	55.15 ± 0.55	38.12 ± 0.34	52.62 ± 0.78	53.12 ± 1.45	48.56 ± 1.45	50.63 ± 0.45
5	89.89 ± 0.95	83.29 ± 0.56	61.91 ± 0.56	40.76 ± 0.88	63.71 ± 0.33	58.63 ± 0.56	56.86 ± 0.48	55.16 ± 0.55
6	90.96 ± 0.46	86.61 ± 0.89	66.81 ± 0.45	44.58 ± 0.33	69.41 ± 0.89	65.36 ± 0.88	61.72 ± 0.34	58.94 ± 0.95
7	93.08 ± 0.60	90.21 ± 1.23	71.67 ± 0.88	48.82 ± 0.45	75.13 ± 0.55	70.93 ± 0.60	66.39 ± 0.56	64.46 ± 0.88
8	94.29 ± 0.34	92.16 ± 0.56	74.56 ± 0.25	51.44 ± 0.77	80.87 ± 0.45	78.03 ± 0.34	72.66 ± 0.66	68.12 ± 0.93
9	99.06 ± 0.45	94.61 ± 0.60	76.09 ± 0.89	56.09 ± 0.23	94.77 ± 0.23	85.36 ± 0.33	76.46 ± 0.90	72.8 ± 0.67
10	—	96.54 ± 0.23	80.28 ± 0.24	61.43 ± 1.33	98.01 ± 0.34	91.73 ± 0.56	82.62 ± 0.55	76.34 ± 0.34
11	—	98.69 ± 0.45	83.4 ± 1.23	65.08 ± 0.33	—	94.55 ± 0.99	89.33 ± 0.78	81.6 ± 0.66
12	—	—	—	71.23 ± 0.45	—	97.13 ± 1.24	92.75 ± 0.88	86.49 ± 0.45

*All above reading are average ± SD, *n* = 6.

**Table 7:** The *in vitro *drug release profiles for the formulations (F9–F18).

Time (hrs)	F9	F10	F11	F12	F15	F16	F17	F18
0	0	0	0	0	0	0	0	0
1	26.34 ± 0.78	23.34 ± 0.96	22.35 ± 0.98	22.78 ± 0.99	23.34 ± 0.78	22.45 ± 0.90	24.34 ± 0.67	22.66 ± 0.88
2	34.27 ± 1.25	36.23 ± 0.67	33.71 ± 0.78	31.41 ± 0.78	38 ± 0.46	36.74 ± 0.67	31.56 ± 0.54	27.89 ± 0.97
3	42.59 ± 0.90	42.19 ± 0.89	36.72 ± 1.02	35.8 ± 0.60	49.04 ± 0.78	44.82 ± 0.78	41.51 ± 0.78	35.81 ± 0.67
4	51.04 ± 0.56	50.83 ± 0.67	43.53 ± 0.67	43.31 ± 0.66	57.2 ± 0.98	51.13 ± 0.65	51.08 ± 1.34	39.45 ± 0.45
5	56.6 ± 0.89	58.22 ± 0.78	46.29 ± 0.69	45.39 ± 0.56	63.3 ± 0.67	62.9 ± 0.67	57.29 ± 1.55	46.78 ± 0.33
6	63.82 ± 1.33	63.37 ± 1.23	49 ± 0.77	47.44 ± 0.88	67.06 ± 0.56	63.36 ± 0.55	60.5 ± 0.78	53.98 ± 0.78
7	67.44 ± 0.56	64.8 ± 0.78	51.58 ± 0.66	53.18 ± 0.73	74.96 ± 0.77	75.08 ± 0.77	65.83 ± 0.56	59.34 ± 0.66
8	70.89 ± 0.34	66.99 ± 1.45	56.52 ± 0.45	55.55 ± 0.67	85.18 ± 0.55	81.15 ± 0.44	77.82 ± 0.55	64.67 ± 0.34
9	76.38 ± 0.45	72.58 ± 0.78	61.84 ± 0.79	59.05 ± 1.67	92.36 ± 0.78	87.63 ± 0.78	81.32 ± 0.88	70.56 ± 0.78
10	81.49 ± 0.68	79.16 ± 0.60	68.24 ± 0.66	62.97 ± 0.78	96.94 ± 0.55	94.53 ± 0.87	88.61 ± 0.98	74.33 ± 0.67
11	86.5 ± 0.56	82.82 ± 0.78	72.31 ± 0.56	69.58 ± 0.23	99.49 ± 0.67	98.91 ± 0.98	95.95 ± 1.56	80.22 ± 0.65
12	93.96 ± 0.95	85.57 ± 0.85	76.59 ± 0.77	72.18 ± 0.56	—	—	99.08 ± 0.88	86.78 ± 0.73

*All above reading are average ± SD, *n* = 6.

**Table 8:** Release kinetics data of the Formulations F1–F18.

S. No	Formulation code	Zero order	First order	Higuchi	Korsmeyer-Peppas	
		*R* ^*2*^	*R* ^*2*^	*R* ^*2*^	*n*	*R* ^*2*^
1	F1	0.6088	0.859	0.725	0.473	0.746
2	F2	0.774	0.988	0.8811	0.466	0.882
3	F3	0.96	0.987	0.974	0.488	0.96
4	F4	0.992	0.956	0.949	0.389	0.925
5	F5	0.988	876	0.992	0.554	0.988
6	F6	0.988	0.917	0.99	0.532	0.988
7	F7	0.985	0.937	0.979	0.533	0.958
8	F8	0.985	0.972	0.995	0.462	0.993
9	F9	0.988	0.905	0.991	0.497	0.989
10	F10	0.972	0.977	0.991	0.459	0.992
11	F11	0.991	0.955	0.992	0.407	0.955
12	F12	0.991	0.974	0.974	0.396	0.961
13	F15	0.986	0.803	0.99	0.543	0.993
14	F16	0.992	0.806	0.984	0.545	0.9885
15	F17	0.992	0.857	0.978	0.549	0.971
16	F18	0.998	0.867	0.977	0.543	0.9734

**Table 9:** Stability study of formulation F17.

Time (months)	Drug content (%)	Floating behavior		*In vitro* drug release at 12th hr
		FLT (min:sec)	Total floating time (hrs)	
Zero	98.07	1:06	>12	99.08
First	98.09	1:05	>12	99.04
Second	99.0	1:06	>12	99.12
Third	99.05	1:05	>12	98.76
